# The TICOPA protocol (TIght COntrol of Psoriatic Arthritis): a randomised controlled trial to compare intensive management versus standard care in early psoriatic arthritis

**DOI:** 10.1186/1471-2474-14-101

**Published:** 2013-03-21

**Authors:** Laura C Coates, Nuria Navarro-Coy, Sarah R Brown, Sarah Brown, Lucy McParland, Howard Collier, Emma Skinner, Jennifer Law, Anna Moverley, Sue Pavitt, Claire Hulme, Paul Emery, Philip G Conaghan, Philip S Helliwell

**Affiliations:** 1 Division of Rheumatic and Musculoskeletal Disease, Leeds Institute of Molecular Medicine, University of Leeds, Leeds LS7 4SA, UK; 2 Clinical Trials Research Unit, Leeds Institute of Molecular Medicine, University of Leeds, Leeds LS2 9JT, UK; 3 Centre for Health Sciences Research, Leeds Institute of Health Sciences, University of Leeds, Leeds LS2 9LJ, UK; 4 Academic Unit of Health Economics, Leeds Institute of Health Sciences, University of Leeds, Leeds LS2 9LJ, UK; 5 NIHR Leeds Musculoskeletal Biomedical Research Unit, Leeds LS7 4SA, UK; 6 Arthritis Research UK Primary Care Centre, Primary Care Services, University of Keele, Staffordshire ST5 5BG, UK

**Keywords:** Psoriatic arthritis, Early psoriatic arthritis, Tight management, Tight control, Intensive management, Standard care

## Abstract

**Background:**

Psoriatic Arthritis (PsA) is estimated to occur in 10-15% of people with psoriasis and accounts for 13% of people attending early arthritis clinics. With an increasing awareness of the poor outcomes associated with PsA and the availability of new effective, but costly, treatments, there is an urgent need to research the optimal treatment for patients with PsA. The aim of the TICOPA study is to establish whether, in treatment naive early PsA patients, “tight control” intensive management with protocol driven therapies and pre-defined objective targets for treatment can improve clinical outcome compared to standard care alone.

**Methods/design:**

TICOPA is a UK multicentre, open-label, randomised controlled, parallel group trial of 206 patients with early PsA. Patients will be randomised on a 1:1 basis to receive either standard care (12 weekly review) or intensive management (4 weekly review) for a period of 48 weeks. Patients assigned to the intensive management group will follow a strict treatment protocol whereby dose continuation/escalation is determined through the objective assessment of the minimal disease activity (MDA) criteria. Patients assigned to the standard care group will have treatment prescribed as felt appropriate by the treating clinician, with no set protocol. The primary objective of the trial is to compare intensive management with standard care in terms of the proportion of patients achieving an ACR 20 response at 48 weeks post-randomisation, in order to determine whether intensive management has superior clinical efficacy. Key secondary outcomes include ACR 50 and 70, PASI 75 and X-ray Van der Heijde score at 48 weeks post-randomisation along with cost-effectiveness at 12, 24 and 28 weeks.

**Discussion:**

The TICOPA trial will provide direct evidence as to whether the use of early and intensive treatment in PsA in routine clinical care leads to an improvement in patients’ disease activity and a reduction in radiological joint damage.

**Trial registration:**

ISRCTN30147736, NCT01106079

## Background

Psoriatic arthritis (PsA) is estimated to occur in 10-15% of people with psoriasis and accounts for 13% of people attending early arthritis clinics [1]. Two thirds of people with PsA suffer progressive joint damage, increasing disability and reduced life expectancy [1-3]. With increasing awareness of these poor outcomes and the availability of new effective but costly treatments, there is an urgent need to research the optimal treatment for patients with PsA.

Research in rheumatoid arthritis (RA) has identified the strong link between inflammation and subsequent joint damage with modern imaging [4]. Subsequently the TIght COntrol of RA (TICORA) study introduced the concept of “tight control” where pre-defined disease activity levels guide therapeutic changes for RA patient management. TICORA demonstrated that tight control of disease resulted in significantly better clinical and radiographic outcomes compared to routine care with no formal therapeutic protocol [5].

There is little research into the link between inflammation and structural joint damage in PsA. Previous cohort studies in established disease have shown that active swollen joints and previous joint damage are predictors of a future increase in the clinically damaged joint count [6] and radiological progression [7]. The potential for imaging studies to aid understanding of the pathogenesis of PsA is well recognised [8], but there has been very little work done using modern imaging in PsA, especially in early disease, to improve mechanistic insight into the disease. There is no research in PsA addressing the concept of tight control of inflammation to reduce joint damage.

Studies in RA using tight control aim for pre-specified low Disease Activity Scores (DAS), and have shown improved disease outcomes [5]. The TICORA study identified the benefit of tight control even before the use of newer biologic agents. This work has led to a shift in the attitudes seen in routine clinical care with further emphasis placed on disease control to prevent further damage. The use of objective outcome measures such as DAS to guide treatment is now becoming routine in clinical care. Research has also continued from this stance with emphasis on early treatment and achieving remission in the care of patients with RA. However, the DAS was developed for RA and is not ideal for use in PsA because it fails to take into account the unique aspects of the disease, and because cut off points for levels of disease activity have never been validated. In RA, there is only one available objective target that does not rely on composite scoring; this is the OMERACT (Outcome Measures in Rheumatology group) definition of minimal disease activity (MDA), which is defined as “a state which is deemed a useful target of treatment by both physician and patient, given current treatment possibilities and limitations” [9]. These criteria are based on the OMERACT core set of domains for RA [9].

A core set of domains for PsA has now also been agreed by OMERACT [10], and new criteria to define disease activity in PsA have now been developed [11]. These criteria for MDA incorporate measures of joint and entheseal inflammation, skin disease, patient reported outcomes and functional ability to assess the patient’s disease activity. These criteria were developed with support from the Group for Research and Assessment of Psoriasis and Psoriatic Arthritis (GRAPPA), an international research consortium for PsA and psoriasis. They have been preliminarily validated using the OMERACT filter with data from independent observational cohorts and interventional trial datasets [12,13]. These data have shown that the MDA criteria represent the concept of MDA accurately, have an ability to differentiate and prognosticate, and are feasible in clinical practice.

From observational data in PsA, it seems likely that there is a link between inflammation and damage; modern imaging should clarify this situation. Suppression of inflammation using new biological therapies has improved outcomes for people with established PsA. However, there are no therapeutic trials assessing treatment in the early stages of disease before significant joint damage has occurred. The therapeutic concept of “tight control” has never been investigated in PsA despite the fact that this is well established as the optimal treatment in early RA. Combining these concepts of early treatment and tight control of inflammation in a therapeutic trial could further elucidate the role of inflammation and establish the optimal treatment for people with PsA.

With the development and validation of the MDA criteria, a disease specific target for treatment has been established in PsA and the use of the principles of tight control that were used in the TICORA study can now be applied to patients with PsA. The aim of the study is to utilise the MDA criteria in a tight control protocol to treat newly diagnosed PsA. The TICOPA study will generate mechanistic evidence to inform whether there is a link between inflammation and damage and provide pertinent knowledge of the benefit of intensive treatment to provide superior clinical care to patients with PsA and a consequent landmark shift in clinical management similar to that observed in RA resulting in part from the TICORA study.

## Methods

### Study aims

The aims of the TICOPA study are to compare the clinical and imaging outcomes over 48 weeks, and safety outcomes over 52 weeks after commencement of therapy in treatment naïve patients with early PsA receiving either “tight control” protocol-led treatment or usual clinical care. The principle hypothesis is that tight control of inflammation in PsA will lead to an improvement in patients’ disease activity and a reduction in radiological damage compared to standard care alone.

#### Primary objective

To compare intensive management to standard care in terms of the proportion of patients achieving an ACR20 response at 48 weeks post-randomisation, in order to determine whether intensive management has superior clinical efficacy.

#### Secondary objectives

To compare intensive management with standard care in terms of additional clinical efficacy outcomes at 24 and 48 weeks, including:

• ACR20 (24 weeks), ACR50 and ACR70

• PASI 50, PASI 75 and PASI 90

• Change in Sharp-van der Heijde Score

• Assessment of cost effectiveness at 12, 24 and 48 weeks

• ASAS 20 and ASAS 40

• Change in enthesitis score

• Change in dactylitis score

• Change in mNAPSI

• Change in HAQ

• Change in other scores (including BASDAI, tender and swollen joint counts, patient and clinician VAS scores)

• MDA score

• Comparison of Quality of Life (QoL),using PsAQoL between intensive management and standard care at baseline, 24 and 48 weeks

• Comparison of safety outcomes over the course of the treatment until 52 weeks

• Comparison of imaging efficacy outcomes including change in Psoriatic Arthritis Magnetic Resonance Imaging Score (PsAMRIS) and ultrasound assessment of disease at 48 weeks in order to assess inflammation and damage (sub-study).

### Study design

The TICOPA trial is designed as a randomised, controlled, parallel group, open label, multi-centre clinical trial of 206 patients with recent onset psoriatic arthritis. Patients will be randomised on a 1:1 basis to receive either standard care (12 weekly review) or tight control (4 weekly review) for a period of 48 weeks, with follow-up of safety up to 52 weeks.

### Study interventions

**Figure 1:**
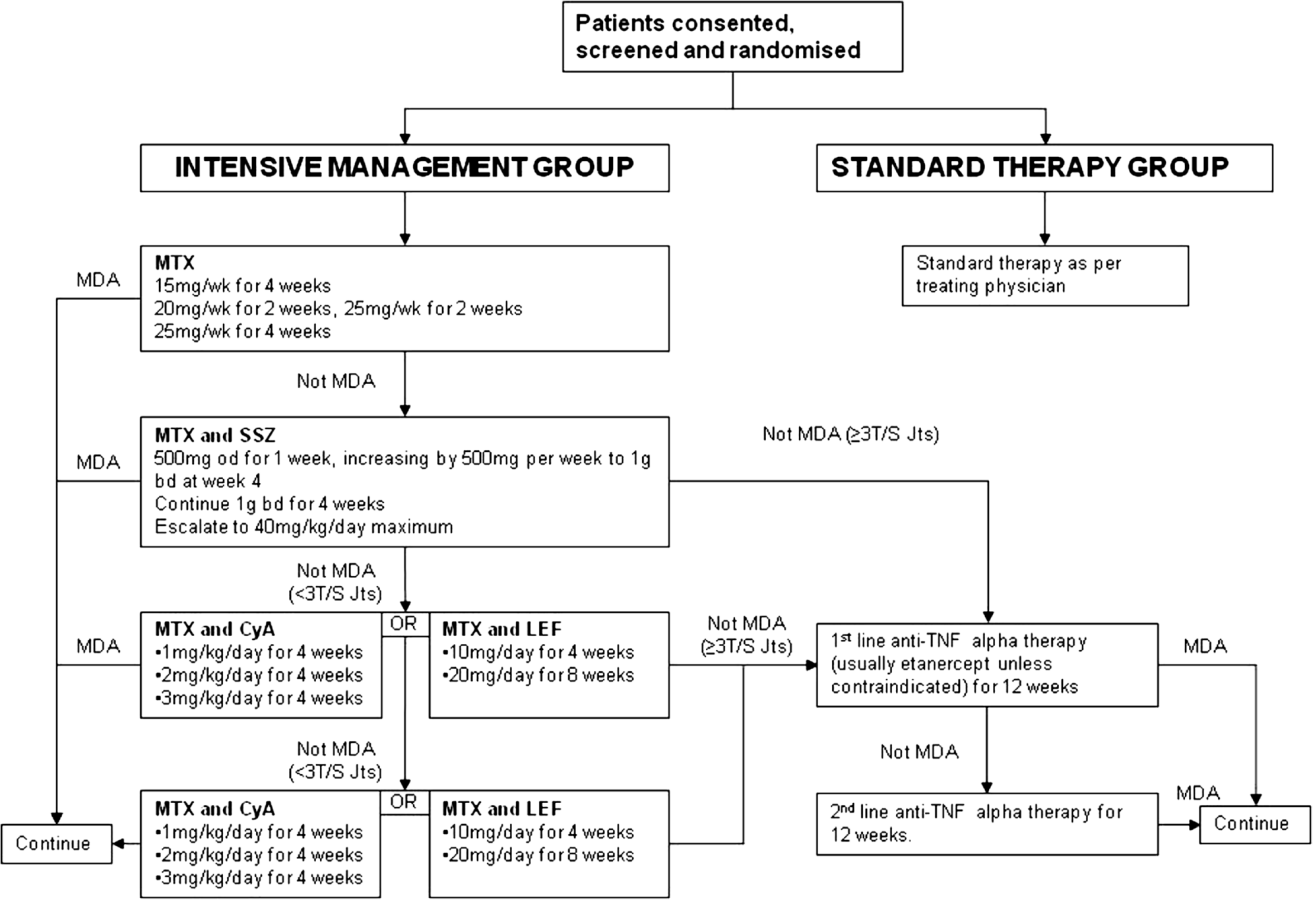
TICOPA Study Flow Diagram.

Patients assigned to the intensive group will be seen every 4 weeks by the study physician. A strict treatment protocol (see Figure 1) is followed for treatment. At each visit, the minimal disease activity (MDA) criteria [11] are assessed. These criteria include assessment of the following: (i) a full 68 tender and 66 swollen joint count, (ii) Psoriasis Area Severity Index (PASI), (iii) enthesitis count, (iv) patient global disease activity VAS, (v) patient pain VAS, and (vi) the Health Assessment Questionnaire (HAQ). Treatment with DMARDs is escalated to the maximum dose according to the protocol in Figure 1 if patients do not achieve the MDA criteria. In the case of drug intolerance, that drug is discontinued and the next step in the protocol is initiated. Any patient who cannot tolerate the maximum dose specified in the protocol due to toxicity or intolerance, is permitted to continue on the highest tolerable dose and then progress to the next step in the protocol if required. Patients achieving these criteria will continue on their current therapy. Intra-articular and intra-muscular steroids are also used in disease control. Patients are offered local joint injections to active joints and/or intramuscular steroid by the physicians if considered appropriate.

Patients randomised to the standard therapy group are treated in a general rheumatology outpatient clinic supervised by a consultant rheumatologist and including trainee rheumatologists working under supervision. These participants are reviewed every 12 weeks or more often if clinically indicated, with no formal measures of disease activity used in clinical decision making. There is no requirement or restriction on prescribing within this arm of the study.

### Recruitment

A total of 206 patients will be recruited over a period of 46 months. Eligible patients will be identified from early arthritis clinics and new referrals to rheumatology clinics at eight hospital sites in the UK. Each site will receive an initiation visit by the member of the research team who will fully train local collaborators in the study.

Patients with a consultant diagnosis of psoriatic arthritis less than two years duration will be recruited. Eligibility for the study is determined at a clinical screening visit with a rheumatologist and research nurse. The study team will ensure that the patient satisfies the study inclusion criteria and does not have any of the exclusion criteria listed in Table 1.

**Table 1:** TICOPA Study inclusion and exclusion criteria

**Inclusion criteria**	
1.	Patients with a diagnosis of psoriatic arthritis by a consultant Rheumatologist with less than 24 months disease duration
2.	Active disease defined by at least one tender or swollen joint or active enthesitis.
3.	Age ≥18 years at the time of signing the informed consent form and either male or female patients.
4.	Patient understands the objectives of the study and is able and willing to sign the Informed Consent Form.
5.	Women of child bearing potential (WCBP) and men whose partners are WCBP must use at least one adequate birth control measure whilst receiving protocol treatment and should continue such precautions after receiving the last dose of protocol treatment as indicated in the relevant SmPC.
6.	Adequate full blood count within 28 days before randomisation:
	a. Haemoglobin count > 8.5 g/dL
	b. White blood count (WBC) > 3.5 × 109/L
	c. Absolute neutrophil count (ANC) > 1.5 × 109/L
	d. Platelet count > 100 × 109/L
7.	Adequate hepatobiliary function within 28 days before randomisation:
	a. ALT and/or AST levels must be within 3 times the upper limit of normal range (ULN) for the laboratory conducting the test.
8.	The patient must be able to adhere to the study visit schedule and other protocol requirements.
**Exclusion criteria**	
1.	Previous treatment for articular disease with disease modifying drugs (DMARDs) including, but not limited to, methotrexate, sulfasalazine, leflunomide.
2.	Women who are pregnant, lactating or planning pregnancy within 6 months of their last dose of protocol treatment.
3.	Use of any investigational agents within 4 weeks or within 5 half-lives of the investigational agent, whichever is longer, prior to randomisation.

Patients will be approached during standard clinic visits and will be provided with a detailed Patient Information Sheet. They will also be provided with a verbal explanation of the trial by the attending medical staff. Patients will be given at least 24 hours to read the information to consider participation. Assenting patients will then be formally assessed for eligibility and invited to provide written informed consent.

Eligible patients participating in the Yorkshire region will be invited to take part in the optional imaging sub-study based in Leeds. Verbal information and a separate Patient Information Sheet will again be provided for the imaging sub-study. Patients will then be invited to provide separate written informed consent for this sub-study.

### Non- randomised patients

Participating research sites will be required to complete a log of all patients, over the age of 18 years with PsA screened for eligibility who are not randomised either because they are ineligible or because they decline participation. Anonymised information collected includes age, gender, ethnicity, reason not eligible for trial participation or for declining participation despite eligibility.

### Randomisation

Following confirmation of eligibility and written informed consent and completion of baseline assessments and questionnaires, patients will be randomised. Randomisation will be performed by an authorised member of staff at the research site. Patients will be randomised on a 1:1 basis to receive either intensive management or standard care. Randomisation will be performed using minimisation, incorporating a random element, via a central 24-hour automated telephone randomisation system based at the Leeds Clinical Trials Research Unit. The dynamic allocation method should ensure treatment groups are balanced for randomising centre and pattern of arthritis (oligoarticular vs polyarticular).

### Pre-treatment investigations

At screening a chest X-ray (CXR) will be performed unless a CXR is available for the patient within the previous 6 months, and this will be checked prior to starting on treatment with methotrexate. If patients are to be prescribed any of the anti-TNF therapies (etanercept, infliximab, adalimumab) then standard screening will apply. Screening for TB will include either a negative Mantoux test or a negative QuantiFERON gold or TB Spot test. In the event of a positive or borderline test, the test is repeated. If this remains borderline or positive, patients are treated with TB prophylaxis (isoniazid 300mg po od and pyridoxine 100mg po od) for a minimum of 2 weeks prior to starting on the TNF therapy. Treatment with isoniazid and pyridoxine is continued for a minimum of 3 months whilst anti-TNF therapy is given.

### Patient follow-up procedures

Patients will be treated in the trial for a period of 48 weeks and followed up for a further 4 weeks. Patients in the intensive management arm will attend the tight control clinic every 4 weeks. For patients in the standard care arm, they will attend a general NHS rheumatology clinic according to local practice dictated by the treating physician, which is usually every 12 weeks.

Patients will be followed up from the baseline assessment to 52 weeks post start of treatment. Follow up assessments will involve a full clinical assessment at 12 weekly intervals to 48 weeks; entailing a physical examination, a full clinical disease assessment, concomitant medical history, and obtainment of safety and efficacy bloods. The follow-up assessment will be performed by a research nurse or metrologist blinded to the allocated treatment group. A final safety assessment will then be conducted at 52 weeks. Where possible, trial clinical assessments will be performed at the same visit as the treatment clinic visits.

Patients who have consented to the imaging sub-study will undergo additional imaging assessments (ultrasound, peripheral MRI and high-field MRI) of peripheral joints and entheses.

Cessation or alteration of treatment regimes will be at the discretion of the clinician or the patient. All patients withdrawn from treatment or prescribed alternative treatment will still attend for follow-up assessments to the end of the study unless the patient withdraws consent for follow-up.

As all therapies are routinely used in the care of PsA, we expect that patients will continue on their medication as long as response has been demonstrated. All patients will be offered continuing follow up in the most appropriate rheumatology clinic at their site after the patient completes the study (a specialised PsA clinic where available).

### Outcome measures

The primary endpoint of the study is achievement of American College of Rheumatology (ACR) 20 response at 48 weeks. The ACR20 is a composite response measure developed for RA and requires an improvement of at least 20% in tender and swollen joint counts and a 20% improvement in three out of five criteria from baseline to 48 weeks: (i) patient global assessment of disease activity (VAS); (ii) physician global assessment of disease activity (VAS); (iii) patient assessment of pain (VAS); (iv) Health Assessment Questionnaire (HAQ), and (v) an inflammatory marker (Erythrocyte Sedimentation Rate (ESR) or C-reactive Protein (CRP)) [14]. This has now been validated as a discriminative outcome measure in PsA [15].

Key secondary endpoints at 48 weeks are: (i) ACR50 and ACR70; (ii) Psoriasis Area Severity Index (PASI) 75; and (iii) modified X-ray Van der Heijde score; and also the cost effectiveness ratio using quality adjusted life year (QALY) outcome measures at 12, 24 and 48 weeks.

The additional secondary endpoints at 24 weeks are (i) ACR20, ACR50 and ACR70 at 24 weeks; (ii) PASI75; and then at 24 and 48 weeks: (iii) ASAS20, ASAS40; (iv) Leeds Enthesitis Index [16,17]; (v) dactylitis score (using the Leeds Dactylitis Instrument); (vi) modified Nail Psoriasis Severity Index (mNAPSI); (vii) HAQ; (viii) PsAQoL; (ix) BASDAI 50; (x) tender and swollen joint counts; (xi) the physician’s assessment of overall disease activity, (xii) the patient’s assessment of global disease activity and pain; (xiii) achievement of minimal disease activity (MDA) criteria; (xiv) radiographic joint damage according to change in modified Sharp/Van der Heijde score, and (xv) overall safety.

For the imaging subgroup, endpoints relating to inflammation (ultrasound synovitis, MRI inflammation) and damage (bone erosion or damage on ultrasound or MRI) will be measured at baseline and at 48 weeks.

### Sample size calculations

The primary outcome of the study is the proportion of patients who achieve an ACR20 response at 48 weeks. Previous data for patients with PsA [18] has shown ACR20 at 3 and 6 months to be 50% and 43% respectively. As it was expected that this patient group had a poorer prognosis we have therefore assumed that the proportion of patients achieving ACR20 in the standard care arm will be approximately 50% at 48 weeks. An absolute difference in ACR20 rates of 20% has been deemed to be a clinically significant difference. Therefore, with 80% power and based on a chi-squared test without continuity at the 2-sided 5% significance level, 93 patients per arm (186 patients in total) are required to detect an increase in ACR20 rates of 20%. To allow for a 10% drop-out rate, 206 patients will be recruited.

### Statistical analysis

An intention-to-treat analysis will be the primary method for analysing and summarising the study data. Patients will be analysed according to the management pathway they were randomised to receive. All formal analyses will be carried out at a 2-sided 5% level of significance.

#### Primary endpoint analysis

For the primary endpoint analysis, treatment groups will be compared by fitting a logistic regression model to response (whether the patient achieved ACR20 at 48 weeks) adjusted for the minimisation factors arthritis classification and centre. Treatment and covariate estimates and odds ratios with corresponding 95% confidence intervals (CIs) will be presented, along with the p-values. In addition the difference in the proportion of patients achieving an ACR20 response will be presented, with corresponding 95% CIs, and compared using a chi-squared test without continuity correction. Multiple imputation will be used to impute missing primary endpoint data. Sensitivity analyses will be performed in order to assess the consistency of the final conclusions and robustness of the ITT population with missing data imputed by multiple imputation.

A repeated measures analysis will also be performed on the primary endpoint in order to assess ACR20 response over the duration of the study. A multi-level repeated measures model will be used to compare ACR20 scores between treatment groups, adjusted for the minimisation factors. Summary statistics at each follow-up time-point will be presented.

#### Key secondary endpoint analysis

Logistic regression adjusting for the minimisation factors, will be used to compare the ACR50, ACR70 and PASI75 between treatment arms at 48 weeks.

All additional secondary endpoints will be summarised descriptively by treatment group and time point. No formal statistical testing will be carried out on these endpoints.

Safety analyses will summarise all serious adverse events, suspected unexpected serious adverse events, adverse events, pregnancies and deaths, and will be presented by treatment group and overall.

### Economic evaluation

The economic evaluation will assess cost effectiveness using within trial incremental cost effectiveness ratios; the costs and benefits of intensive management to the costs and benefits of usual care over trial duration. Analysis will use overall survival and quality adjusted life years (QALYs) outcome measures. Sensitivity analysis of the incremental cost effectiveness ratio will be undertaken. A scatter plot on the cost effectiveness plane, the 95% cost effectiveness elipse and the cost effectiveness acceptability curve will be presented.

## Discussion

PsA is a common and disabling disease but it has received less attention than RA in both clinical and translational research. Evidence from RA has identified the link between inflammation and subsequent joint damage, leading to an emphasis on early aggressive treatment to reduce joint inflammation causing less joint damage and improved outcome. However, the relationship between inflammation and damage has not been well studied in PsA. Observational studies have shown that swollen joints often subsequently become damaged on X-rays and studies using new, highly effective anti-inflammatory therapies in PsA have shown improvement in disease activity in association with a reduction in levels of joint damage over time. Observational cohort data from Toronto has shown that patients presenting to clinic with less than two years disease duration fared better than those presenting later in terms of ongoing progressive joint damage, despite controlling for baseline joint damage [12]. Together, this body of research suggests that a similar ‘tight control’ treatment strategy to that applied in RA may be successful in improving outcomes in PsA. The TICOPA study aims to provide evidence-based optimal treatment for people diagnosed with PsA.

The effect of treating people with more intensive therapies to ensure that their joint inflammation is minimised, will be assessed. It is expected that joint damage will be reduced further in those patients treated intensively, therefore preventing disability. This therapeutic trial will provide direct evidence to inform the use of early and intensive treatment in PsA to ensure improvement in patients’ disease activity and a reduction in radiological joint damage. In addition, the detailed imaging assessments used during the study aims to advance our mechanistic understanding of the relationships between inflammation, damage and bony proliferation in PsA.

Evidence of the benefit of intensive treatment from the TICOPA study should cause a similar shift in routine practice to that seen in RA to provide superior clinical care to patients with PsA.

### Time plan for the TICOPA study

Patient recruitment began in May 2008 at one site (Leeds Teaching Hospital NHS Trust); a total of 206 patients have been recruited across eight centres, and the trial closed to recruitment on 21^st^ March 2012. The trial is currently in follow-up until March 2013.

#### Ethical consideration

Ethical and governance approval for this study has been obtained from the Northern and Yorkshire Research Ethics Committee (ref 07/H0903/72) and the Leeds Teaching Hospitals NHS Trust respectively. The trial progress is monitored by an independent Data Monitoring and Ethics Committee (DMEC) and Trial Steering Committee (TSC).

## Abbreviations

ACR: American college of rheumatology; ADR: Adverse drug reaction; AE: Adverse event; AMRIS: Arthritis magnetic resonance imaging score; ANA: Anti-nuclear antibody; ASAS: Assessment in ankylosing spondylitis; AST: Aspartate aminotransferase; BASDAI: Bath ankylosing spondylitis disease activity index; BNF: British national formulary; BSR: British society of rheumatology; CI: Chief investigator; CRF: Case report form; CRP: C-reactive protein; CTCAE: Common terminology criteria for adverse events; CTRU: Clinical trials research unit; CXR: Chest x-ray (radiograph); DAS: Disease activity score, 4 variable using either ESR or CRP; DMARD: Disease modifying anti-rheumatic drug; DMEC: Data monitoring and ethics committee; dsDNA Ab: Double stranded DNA antibodies; EUC: Electrolytes, urea & creatinine; FBC: Full blood examination; GCP: Good clinical practice; GDA: Global assessment of disease activity; GH: Patient‘s general health visual analogue scale (VAS); GRAPPA: Group for assessment of psoriasis and psoriatic arthritis; HAQ: Health assessment questionnaire; Hb: Haemoglobin; IA: Intra-articular; IB: Investigator brochure; IMP: Investigational medicinal product; IV: Intravenous; LDCI: Leeds dactylitis instrument; LEI: Leeds enthesis index; LFT: Liver function tests; MASES: Maastricht ankylosing spondylitis enthesitis scale; MDA: Minimal disease activity; MEI: Mander enthesitis index; Mg: Milligrams; mNCA: Model non-commercial agreement; MREC: Main research and ethics committee; MRI: Magnetic resonance imaging; MTX: Methotrexate; mNAPSI: Modified nail psoriasis severity index; NAPSI: Nail psoriasis severity index; NICE: National institute for health and clinical excellence; NSAID: Non-steroidal anti-inflammatory drug; NSF: National Psoriasis Foundation; OD: Once daily; PASI: Psoriasis area severity index; Phys VAS: Physical visual analogue scale; GDA: Physician global assessment of disease activity; OMERACT: Outcome measures in rheumatology clinical trials; PI: Principal investigator; PO: Per os - Oral; PsARC: Psoriatic arthritis response criteria; PsAQoL: Psoriatic arthritis quality of life; PsAMRIS: Psoriatic arthritis magnetic resonance imaging score; PSSRU: Personal social services research unit; Pt VAS GDA: Patient global assessment of disease activity; PV: Plasma viscosity; QoL: Quality of life; RBC: Red blood cell (count); REC: Research ethics committee; SAE: Serious adverse event; SAR: Serious adverse reaction; SC: Subcutaneous; SGOT: Serum glutamic-oxaloacetic transaminase; SmPC: Summary of product characteristics; SUSAR: Suspected unexpected serious adverse reaction; TB: Tuberculosis; TNF: Tumour necrosis factor; ULN: Upper limit of normal; US: Ultrasound; VAS: Visual analogue scale; WBC: White blood count; WCBP: Woman of child bearing potential; WCC: White cell count; WHO: World health organization; Wk: Week.

## Competing interests

Laura Coates has received research funding and/or honoraria from Abbott, Janssen, MSD, Pfizer and UCB. The authors declare that they have no competing interests.

## Authors’ contributions

PH and LC conceived the study, and participated in its design and coordination. LC also led the drafting of the manuscript. NNC was responsible for the coordination of the study and led the drafting of the protocol. SRB participated in the design of the study protocol with responsibility for the design of the statistical analysis of the study. SB is responsible for providing statistical supervision for the study. LMcP has produced the statistical analysis plan and is responsible for conducting the statistical analysis and reporting of the study. SP participated in the overall design of the study. AM participated in the coordination of the study. CH participated in the design of the study protocol, with particular responsibility for the health economics. HC and JL are primarily responsible for the acquisition of clinical data. ES was responsible for the overall management of the study and for the drafting of the protocol. PE and PGC participated in the design of the study protocol. All authors were involved in the protocol drafting and contributed to and approved the final manuscript.

## Pre-publication history

The pre-publication history for this paper can be accessed here:

http://www.biomedcentral.com/1471-2474/14/101/prepub
